# Indications to Hospital Admission and Isolation of Children With Possible or Defined Tuberculosis: Systematic Review and Proposed Recommendations for Pediatric Patients Leaving in Developed Countries: Erratum

**DOI:** 10.1097/01.md.0000480683.34568.fd

**Published:** 2016-02-08

In the article “Indications to Hospital Admission and Isolation of Children With Possible or Defined Tuberculosis Systematic Review and Proposed Recommendations for Pediatric Patients Leaving in Developed Countries”,^1^ which appeared in Volume 94, Issue 50 of *Medicine*, the title contained an incorrect word. The word “Leaving” should have read “Living”. The article has since been corrected online.
